# Genomic Uracil and Aberrant Profile of Demethylation Intermediates in Epigenetics and Hematologic Malignancies

**DOI:** 10.3390/ijms22084212

**Published:** 2021-04-19

**Authors:** Ryszard Olinski, Geir Slupphaug, Marek Foksinski, Hans Einar Krokan

**Affiliations:** 1 Department of Clinical Biochemistry, Faculty of Pharmacy, Collegium Medicum in Bydgoszcz, Nicolaus Copernicus University in Toruń, 85-092 Bydgoszcz, Poland; 2 Department of Clinical and Molecular Medicine, NTNU Norwegian University of Science and Technology, NO-7491 Trondheim, Norway; 3 Clinic of Laboratory Medicine, St. Olavs Hospital and PROMEC Proteomics and Modomics Experimental Core at NTNU and the Central Norway Regional Health Authority, NO-7491 Trondheim, Norway

**Keywords:** DNA methylation, DNA modifications, epigenetics, DNA repair

## Abstract

DNA of all living cells undergoes continuous structural and chemical alterations resulting from fundamental cellular metabolic processes and reactivity of normal cellular metabolites and constituents. Examples include enzymatically oxidized bases, aberrantly methylated bases, and deaminated bases, the latter largely uracil from deaminated cytosine. In addition, the non-canonical DNA base uracil may result from misincorporated dUMP. Furthermore, uracil generated by deamination of cytosine in DNA is not always damage as it is also an intermediate in normal somatic hypermutation (SHM) and class shift recombination (CSR) at the *Ig* locus of B-cells in adaptive immunity. Many of the modifications alter base-pairing properties and may thus cause replicative and transcriptional mutagenesis. The best known and most studied epigenetic mark in DNA is 5-methylcytosine (5mC), generated by a methyltransferase that uses SAM as methyl donor, usually in CpG contexts. Oxidation products of 5mC are now thought to be intermediates in active demethylation as well as epigenetic marks in their own rights. The aim of this review is to describe the endogenous processes that surround the generation and removal of the most common types of DNA nucleobase modifications, namely, uracil and certain epigenetic modifications, together with their role in the development of hematological malignances. We also discuss what dictates whether the presence of an altered nucleobase is defined as damage or a natural modification.

## 1. Introduction

The DNA of all living cells undergoes continuous structural and chemical alterations resulting from fundamental cellular metabolic processes and reactivity of normal cellular metabolites and constituents. Examples include base alterations caused by reactive oxygen species (ROS) from mitochondrial respiration, incorporation of non-canonical dNTPs in DNA replication, nonenzymatic DNA methylation by the cellular methyl donor S-adenosylmethionine (SAM), as well as hydrolytic decay of DNA in the aqueous environment of the cell. The resulting subtle modifications of DNA include oxidized bases, aberrantly methylated bases, and deaminated bases, the latter largely uracil from deaminated cytosine. In addition, the non-canonical DNA base uracil may result from misincorporated dUMP, since dUTP is a normal intermediate in the biosynthesis of dTTP [1,2]. Many of the modifications alter base-pairing properties and may thus cause replicative and transcriptional mutagenesis. In addition, they may alter the binding of DNA-interacting proteins and increase or decrease the binding of transcription factors to their targets [3,4].

**Figure 1:**
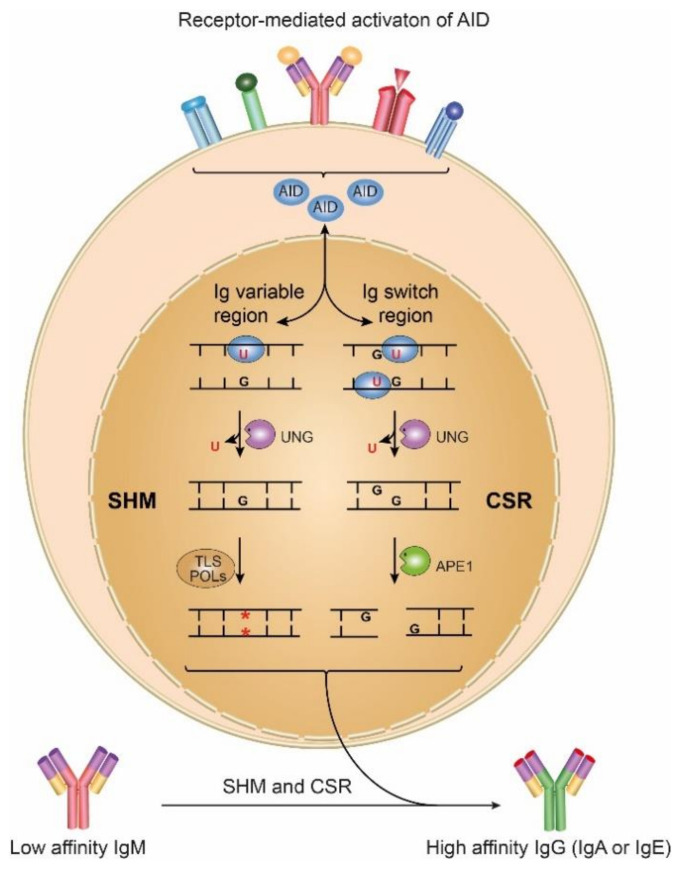
AID initiates SHM and CSR by deaminating cytosine to uracil in *Ig* variable and switch regions, respectively. In SHM, C → T transitions can be induced by direct replication over the uracil. A full mutation spectrum can be achieved after UNG-mediated excision of uracil and replication over the abasic site by error-prone translesion polymerases, often combined with MMR. In CSR, UNG-mediated removal of uracil followed by strand cleavage at the abasic sites by APE1, leads to DSBs in *Ig* switch regions. *Ig* variable segments can then be joined to novel constant region segments by non-homologous end-joining (NHEJ) to form antibodies with novel effector functions.

The best known and most studied epigenetic mark in DNA is 5-methylcytosine (5mC), generated by methyltransferases that use SAM as a methyl donor, usually in CpG contexts. The oxidation products of 5mC are now thought to be intermediates in active demethylation, as well as epigenetic marks in their own rights. Furthermore, uracil generated by the deamination of cytosine in DNA is not always damage as it is also an intermediate in normal somatic hypermutation (SHM) and class shift recombination (CSR) at the *Ig* locus of B-cells in adaptive immunity. The seminal discovery of activation-induced cytosine deaminase (AID, AICDA) some two decades ago was the clue to understanding the mechanisms of SHM and CSR (Figure 1), although AID was originally thought to be an RNA deaminase [5].

AID is expressed in antigen receptor-activated germinal center B-cells and is thought to act on single-stranded DNA regions generated during transcription by RNA polymerase II. AID deaminates cytosine in single-stranded DNA to uracil, initiating the process that results in mutations in variable and switch regions of *Ig* genes at a rate almost a million times higher than spontaneous mutations rates in somatic cells. This process also requires one of the uracil DNA N-glycosylase (UNG)-proteins encoded by the *UNG* gene (Table 1) to generate abasic sites as intermediates, as reviewed [6]. However, this dual function in DNA repair and adaptive immunity is a two-edged sword, as outlined in the text.

The aim of this review is to describe the endogenous processes that surround the generation and removal of the most common types of DNA nucleobase modifications, namely, uracil and certain epigenetic modifications, together with their role in the development of hematologic malignances. We have mostly focused on multiple myeloma (MM), which is the second most common hematologic malignancy. Moreover, MM is a progressive disease often preceded by an asymptomatic stage, monoclonal gammopathy of undetermined significance (MGUS), and by an intermediate stage, smoldering multiple myeloma (SMM). This provides a unique opportunity to study the multistep driver events of malignant evolution [9].

## 2. DNA Glycosylases—More Than Initiators of Base Excision Repair (BER)

**Figure 2:**
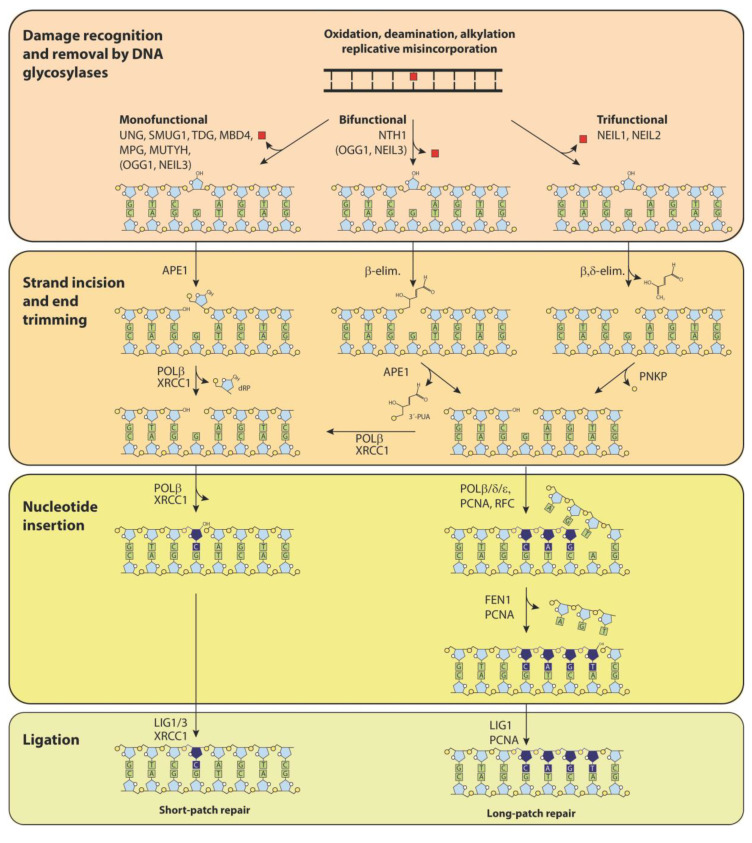
Subpathways in base excision repair (BER). BER takes place by either short patch repair, in which only one nucleotide is inserted (track on the left) or long patch repair, where several nucleotides are inserted (tracks on the right). The principal steps in BER include the following: 1. Damage recognition and base removal by a DNA glycosylase; 2. Strand incision and end trimming; 3. Nucleotide insertion; 4. Ligation of DNA ends. Monofunctional DNA glycosylases remove the damaged base, leaving an abasic site that is cleaved by AP-endonuclease APE1. DNA polymerase β removes deoxyribose phosphate (dRP) and inserts a single nucleotide. Bifunctional DNA glycosylases remove the base and cleave the DNA strand by β-elimination, then APE1 releases the 3′-PUA (3′-phospho unsaturated aldehyde). Trifunctional DNA glycosylases in addition remove the unsaturated aldehyde by δ-elimination, leaving 3′ and 5′ phosphate ends. PNKP (polynucleotide kinase/phosphatase) removes the 3′-phosphate. Note that short patch repair largely uses specialized enzymes in the downstream steps, whereas long patch repair involves several DNA replication proteins. Furthermore, BER of genomic uracil may take place both by short patch and long patch BER [7].

Many modified nucleobases may have profound functional consequences for the cell. Their processing may be very different depending on whether the alteration is read as a DNA “damage” that must be repaired or a DNA “modification” that specifies a function. Damaged bases in DNA are most frequently processed by the BER pathway (Figure 2), which is initiated by one of at least 11 different DNA glycosylases that recognize and remove damaged or inappropriate bases in DNA, thus preventing mutagenicity and cytotoxicity from such changes.

These enzymes initiate BER by removing the damaged or misplaced base, leaving an abasic site as the first intermediate in the repair process [7]. However, it is now known that DNA glycosylases have “non-canonical” roles in adaptive immunity, regulation of gene expression, and other processes. For some of these processes, but not all, the DNA glycosylase activity is required. It is likely that our understanding of the roles of these proteins, as well as their DNA base substrates, is less complete than previously thought. This is elucidated by the diverse functions of DNA glycosylases UNG2, thymine DNA glycosylase (TDG), methyl-CpG binding domain protein 4 (MBD4), single-strand selective monofunctional uracil DNA glycosylase 1 (SMUG1), and 8-oxoguanine DNA glycosylase 1 (OGG1). Whereas UNG2 is required for adaptive immunity, TDG is involved in gene regulation and active demethylation, and MBD4 is a methyl binding protein involved in gene regulation. SMUG1 is a backup for UNG2 in genomic uracil removal and the main enzyme for in vivo removal of 5-hydroxymethyl uracil (5hmU) in DNA [10]. Importantly, it also has a role in RNA quality control, as reviewed [11]. OGG1 binds to the oxidation product 8-oxoguanine (8-oxoG) in gene promoters and recruits transcription factor NF-κB to promoters [12]. In gene promoters harboring potential G-quadruplexes, the removal of 8-oxoG by OGG1 yields an abasic site that locally melts the duplex and allows the binding of APE1. Instead of mediating strand cleavage, this enhances gene expression, thereby suggesting an epigenetic function of 8-oxoG [13]. Very recently, OGG1 was also shown to promote histone H4 dimethylation under oxidative stress, thereby enhancing *c-Myc* transcription [14]. Mammalian Nei-like DNA glycosylases NEIL1, 2, and 3, as well as OGG1 and NTHL1 (NTH1), recognize and remove oxidized bases from DNA. They have, in part, overlapping substrate preferences and have generally been assumed to serve as back up enzymes for each other in the removal of oxidized bases. They may be particularly important in the brain, which has a high number of ROS-producing mitochondria, making this organ prone to deleterious effects of oxidized DNA bases. Surprisingly, neither individual NEIL mouse knockouts, double knockouts, nor triple knockouts displayed changes in survival, telomere length, spontaneous mutation rates, or tumor formation. In addition, mouse embryonic fibroblasts (MEFs) from these knockout mice did not show increased sensitivity to hydrogen peroxide or potassium dichromate exposure [15]. However, NEIL1-knockouts revealed reduced memory retention as tested in a water maze, whereas several other cognitive as well as motor functions were normal [16] (reviewed in [17]). Furthermore, brain damage after carotid artery occlusion and reperfusion was more severe in NEIL1 knockouts, as compared to wildtype mice [16]. Similar results after induced brain damage have also been observed in OGG1 [18] and UNG knockout mice [19]. It is unknown whether the brain-protective functions of these enzymes depend on the DNA glycosylase function. Expression of NEIL3 is largely restricted to hippocampal stem cells, and this DNA glycosylase appears to have an important function in neurogenesis and hippocampal function in adult mice. *NEIL3*^−/−^ mice displayed impaired learning ability and memory retention, as well as reduced anxiety (reviewed in [17]). Although the mechanisms behind these altered brain functions remain uncertain, it is tempting to suggest that not just BER but also an epigenetic role and a role in DNA replication of the glycosylases may be involved. There is certainly also a strong precedence for the epigenetic roles of uracil-DNA glycosylase TDG [20], and possibly UNG, by removing 5-carboxylcytosine from DNA in the active demethylation process [21].

## 3. The Mutagenicity of U:G Mismatches and U:A Pairs in DNA

Mammalian DNA polymerases utilize dUTP and dTTP for DNA synthesis with equal efficiency [22]. The incorporation of uracil instead of thymine results in inappropriate U:A base pairs in DNA. Uracil in DNA may also arise as a consequence of spontaneous deamination of cytosine, leading to U:G mismatches that are directly mutagenic by leading to C→T transition mutations upon DNA replication [23]. The contribution of U:G mismatches to overall mutagenicity would then depend on the number of mismatches generated and the efficiency of their repair. The number of spontaneous cytosine deaminations is estimated to be in the range of 100–300 per day in mammalian cells [24] and would be expected to be manageable for the BER and mismatch repair systems. However, repair efficiency may depend both on the sequence [25] and chromatin context [26]. Importantly, enzymatic cytosine deamination by AID and other members of the APOBEC protein family has been a gamechanger in the understanding of genomic uracil in mutagenicity and cancer development, as discussed below.

U:A base-pairs are not directly mutagenic. However, their presence may lead to mutations when uracil is excised by uracil DNA glycosylase, leaving a cytotoxic and mutagenic abasic site. The mutagenicity of abasic sites resulting from incorporation of dUMP and removal of genomic uracil by UNG was demonstrated using yeast mutants defective in dUTPase and suppression of mutagenicity by simultaneous inactivation of Ung [27,28]. The repair of abasic sites in yeast in vivo can be carried out using three repair pathways to avoid mutagenicity [29]. The mutation frequency of U:A pairs or abasic sites in vitro using extracts of mammalian cells was found to be in the order 1 per 10^4^ abasic sites [30,31]. Although these results indicate that the repair process is quite accurate, they also suggest that AP sites may contribute significantly to mutagenicity if a high number of such sites are generated per round of replication.

The rate of incorporation of dUMP in DNA replication, or in DNA repair processes, most likely depends on the dUTP to dTTP ratio, which displays surprisingly high variation between different cell types, both normal and malignant. In addition, drugs or knockdown of enzymes required for biosynthesis of dTTP increase dUTP/dTTP ratios several-fold. In unperturbed proliferating cancer cells in culture, the dUTP/dTTP ratio was found to be in the range of 0.01–0.05. However, in slowly proliferating or nonproliferating cells this ratio may be in the range of 0.5–1.9 and in human primary macrophages as high as 60 (reviewed in [32]). The extremely high levels of dUTP/dTTP in macrophages may reflect the assumed role of dUTP incorporation and viral DNA degradation by UNG in the defense against retroviral infection [33]. This elucidates yet another function of UNG proteins. Even ratios in the range of 0.01–0.05, as observed in unperturbed tumor cells, are actually very high. Unless some so-far-unknown mechanism exists to prevent incorporation of dUMP, these ratios would suggest that millions of U:A pairs are generated in each round of DNA replication. This is orders of magnitude higher than the calculated number of spontaneous cytosine deaminations per day, suggesting that misincorporation by replicative DNA polymerases is the dominant source of genomic uracil. Furthermore, efficient removal of genomic uracil most likely implies that the number of abasic sites generated essentially equals the number of dUMP residues incorporated. Nuclear UNG2, present in replication foci, is responsible for the fast removal of incorporated uracil, but *UNG*^−/−^ mice apparently replicate their DNA and grow normally and display no overt phenotype until late in life. Furthermore, isolated nuclei from UNG knockout cells [34], as well as nuclei from HeLa cells treated with the UNG inhibitor protein Ugi [35], do eventually remove incorporated dUMP, albeit slowly, presumably by SMUG1.

## 4. Methylation and Demethylation of DNA

**Figure 3:**
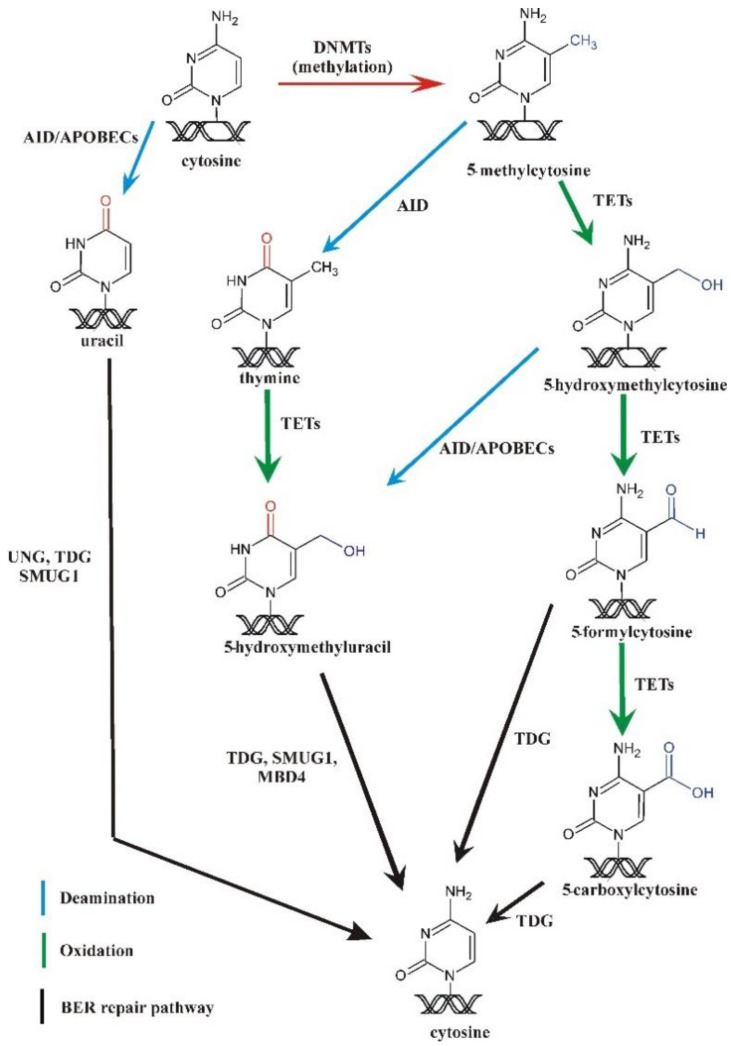
Cytosine methylation and active demethylation pathway. 5mC is formed in DNA methyltransferase (DNMT)-catalyzed reaction. The mechanisms of active 5mC demethylation include involvement of ten-eleven translocation (TET) proteins in oxidation of 5mC.

DNA methylation occurs mainly at C5 of cytosine and is involved in a range of diverse biological processes, including gene expression, which, in turn, has a profound impact on cellular identity and organismal fate. Methylation is catalyzed by DNA methyltransferases (DNMTs), which can be classified as de novo methyltransferases (DNMT3A and 3B in mammals) [36] and maintenance methyltransferases (DNMT1 in mammals). Moreover, proper erasure of DNA methylation is crucial for reprogramming and for maintenance of pluripotency during embryonic development [37,38]. Traditionally, DNA methylation was thought primarily to be erased via passive dilution, but research through the last two decades has revealed that 5mC in cellular DNA is highly dynamic. Ten years ago, two reports [39,40] demonstrated that 5mC can be enzymatically oxidized to 5hmC and that genomic DNA contains up to 0.6% of 5hmC [41]. This reaction is catalyzed by a family of ten-eleven translocation proteins (TET 1, 2, and 3 enzymes), and the oxidation reaction can proceed further to generate 5-formylcytosine (5fC) and 5-carboxycytosine (5caC) (Figure 3); 5fC and 5caC may subsequently be recognized and removed by BER to complete the process of active DNA demethylation (reviewed in [37,42]). Recently, 5-hydroxymethyluracil (5hmU) was shown to be generated also from thymine via a TET catalyzed reaction [43]. An alternative route of demethylation has been proposed in zebrafish based on AID-mediated deamination of 5mC to thymine. This would generate a G:T mismatch that in turn is repaired by MBD4 [44].

Finally, it has been suggested that canonical long patch BER as well as non-canonical mismatch repair may erase epigenetic methylation indirectly during the fill-in phase and that this may be initiated by AID-mediated deamination of unmodified cytosines [45,46,47]. Accumulated evidence suggests, however, that the most plausible mechanism of active 5mC demethylation in humans is via TET oxidation of 5mC to 5hmC and the latter further to 5fC and 5caC. BER initiated by TDG [48,49] and NEIL1 and NEIL2 [50,51] then replaces these modified bases with cytosine to demethylate DNA (reviewed in [37,52]). Nevertheless, a non-oxidative component of active 5mC demethylation was recently demonstrated in mouse pluripotent cells, although the precise mechanism involved could not be identified [53].

5hmC is thus a key player in active demethylation. Some data further suggest that it is not only a demethylation intermediate but also an epigenetic mark on its own [54]. It has also been proposed that 5hmC may serve as biomarker of cancer risk [55], diagnosis, and treatment (reviewed in [56]).

## 5. Genomic Uracil, Uracil Repair Deficiency and B-Cell Malignancy

Whereas mouse embryonal fibroblasts (MEFs) from UNG knockout mice and human lymphoblastoid cells display a 5–10-fold increase in genomic uracil [57], mouse organs of UNG knockouts generally display only some two-fold increase, and SMUG1 knockouts no significant increase [10]. However, UNG/SMUG1 double knockouts display dramatic increases in genomic uracil in most, but not all, mouse organs. Thus, UNG2 and SMUG1 are clearly backup enzymes for each other, although SMUG1 also has a distinct function in the removal of 5-hydroxymethyluracil in vivo [10]. Furthermore, UNG knockout mice had only a 1.4–1.8-fold increase in mutation rates and no general increase in cancer frequencies [34]. However, UNG knockouts displayed a 22-fold increase in malignancies of B-cell lymphomas late in life, preceded by lymphatic hyperplasia [58]. This B-cell specificity is likely associated with the function of UNG in antibody diversification in B-cells. In agreement with this, UNG-deficient mice were defective in class switch recombination (CSR) [59]. Subsequently, humans carrying inactivating mutations in the UNG gene were shown to be defective in CSR and have skewed somatic hypermutation (SHM) as well as lymphoid hyperplasia. The low number of known patients carrying inactivating mutations in the *UNG* gene so far precludes an evaluation of the possible development of lymphomas caused by *UNG* mutations in humans [60]. However, interindividual variation in UNG activity in lymphocytes is high and may be a more likely risk factor [61].

Furthermore, it has become apparent that UNG may also contribute to the development of B-cell malignancy, together with AID. Chromosomal translocations involving immunoglobulin switch regions is a hallmark of B-cell malignancies. In a mouse model, the generation of such translocations required both AID and UNG [62]. Furthermore, in a mouse model for BCL-6-driven diffuse large B-cell lymphomas (DLCBL), UNG2-deficiency actually protected against tumor development driven by AID [63]. Thus, UNG2 may under some circumstances protect against the development of B-cell malignancy while contributing to malignancy under other conditions. The apparent paradox that UNG contributes to malignancy when expression of BCL6 is enforced may reflect the complexity of the process. Thus, the generation of mutagenic U:G mismatches and abasic sites by AID and UNG is inherently risky and must normally be well balanced. The central role of AID in the development of B-cell malignancies was supported by the observation that such cancers carry a distinct mutational AID signature, while many other types of human cancer did not [64].

## 6. Activation-Induced Cytosine Deaminase as a Source of Uracil in DNA: A Link to Genome Instability

Replication across U:G mispairs generated by AID directly generates C→T transition mutations observed in SHM. A lack of UNG increases the fraction of C→T mutations, as expected [59]. Generally, processing of uracil generated by AID is thought to specifically require recruitment of the nuclear form UNG2. However, it was recently found that, at least in mice, a quantitatively minor, longer variant of UNG1 also fully supports CSR. Selective knockout of either UNG1 or UNG2 did not abrogate CSR, whereas knockout of both forms fully eliminated CSR [8]. In the antibody diversification process, UNG generates abasic sites that provide noninstructional lesions as a basis for SHM, whereas closely spaced abasic sites in switch regions can be converted to strand breaks required for CSR. Thus, the UNG enzyme deficiency may on the one hand be responsible for immunological dysregulation and on the other hand may lead to development of B-cell lymphomas in the case of AID mistargeting.

One intriguing question was whether the unprecedented level of mutation is restricted to regions of the antibody genes or if expressed genes in activated B cells other than *Ig* genes also undergo high rates of mutation. Subsequent studies have demonstrated that AID deaminates cytosine moieties in 25% of the expressed genes of mouse germinal center B cells [6]. However, the mutation rate of these genes is 20–40-fold lower than that of the *Ig* loci, but still much higher than the spontaneous mutation rate [6]. It was shown that there are two levels of genome protection: (i) selective targeting of AID and (ii) a safety net of high fidelity DNA repair mechanisms that removes uracil generated by AID just like uracil formed by other processes [6]. Dysregulation of these processes may lead to malignant transformation.

## 7. Aberrant AID Expression and Malignant Development

Aberrant AID expression, which can lead to genome-wide mutations in genes other than *Ig* genes and/or in non-lymphoid cells, may contribute to genetic changes resulting in malignancy. Constitutive AID expression in transgenic mice causes the development of lymphomas [65]. These animals also developed tumors in several other tissues [66,67,68]. Furthermore, a variety of human tumors have AID-generated mutations in key oncogenes (e.g., *MYC*) and the tumor suppressor gene *TP53* [6]. Aberrant AID expression was also reported in human B-cell non-Hodgkin’s lymphoma [69]. Moreover, AID-dependent activation of *MYC* gene induces MM in a mouse model of this malignancy [70].

Besides AID, there is another enzymatic source for the presence of uracil in DNA, namely, apolipoprotein B editing complex (APOBEC) enzymes. Of note, it was demonstrated that APOBEC upregulation was responsible for elevated uracil levels in cellular DNA and increased mutation rates in breast cancer cell lines [71]. APOBEC3B upregulation was found in a majority of human breast tumors, while it was barely detectable levels in normal cells. Moreover, APOBEC3B overexpression correlated with (i) a doubling in the tumor genomic mutation loads and (ii) inactivation of the tumor suppressor gene *TP53*, strongly suggesting that it may be an early tumor-initiating event [71] (reviewed in [72]). Of note, aberrant APOBEC activity also appears to have relevance in predicting high-risk MM [73].

## 8. Involvement of AID and APOBEC Enzymes in MM Development

Recent studies concerning driver events in MM strongly suggest that aberrant AID activity, associated with a specific mutational landscape, is mostly involved in the tumor initiation, while later phases of MM development are linked to an APOBEC mutational process [74,75]. Moreover, mutational signatures of AID/APOBEC, together with those linked to aging and DNA repair deficiency account for ~80% of all mutations in MM [73]. However, the molecular mechanisms giving rise to these mutations are yet to be fully elucidated. There is no doubt that the direct product of AID and APOBEC activity is uracil formation in DNA (via cytosine deamination). Even if we assume that most newly formed uracil moieties occur in the non-coding part of the genome, it should increase the global mutation burden. Sequencing studies of mutational landscape in MM showed a huge heterogeneity, failing to identify clear molecular pathophysiologic event(s) responsible for the disease development. This, in turn, pointed to an unspecific random action, such as aberrant uracil formation, as an important risk factor in MM development. Thus, it is possible that the determination of uracil may be a good marker of MM development.

## 9. Is There a Link between Active Demethylation and Malignant Transformation?

Generally, methylation of CpG islands limits binding of transcription factors at gene promoters. Global CpG hypomethylation is known to occur in human cancers and precancerous conditions, although hypermethylation is also observed at distinct loci [76] (reviewed in [77]). This has led to suggestions that hypomethylation enhances genetic instability and malignant transformation, e.g., by the activation of oncogenes [78] and increased genomic rearrangements due to activation of transposable elements that are normally silenced within heterochromatin [79]. Genomic hypomethylation may be caused by decreased activity of DNMTs as well as increased oxidative or non-oxidative demethylation, and there is evidence in support of both mechanisms as drivers of malignant transformation. In humans, mutations in DNMT3B have been found in several hematological malignancies including cutaneous T-cell lymphomas and B-cell lymphomas [80]. Moreover, Dnmt3b-deficient mice display genome-wide hypomethylation, increased expression of several oncogenes, and develop T-cell lymphomas and chronic lymphocytic leukemia [81]. TET1 was identified as a fusion partner of the mixed-lineage leukemia (MLL) gene from the breakpoint of chromosomal translocation in acute myeloid leukemia (AML) [82], and loss of TET2 is strongly associated with myelodysplastic syndromes, myeloproliferative neoplasms, and myeloid leukemias [83].

As mentioned above, recent evidence suggests that TETs may also catalyze synthesis of 5hmU from thymine. According to Pfaffeneder et al. [43], the level of 5hmU changes during the course of epigenetic reprogramming of the cell, following the same pattern as other products of TETs: 5hmC, 5caC, and 5fC. This implies that 5hmU may have an epigenetic function, similar to that of other products of active DNA demethylation.

## 10. What Has Been Learned about 5hmC in Tumor Development?

Recent works have shown that 5hmC is profoundly reduced in many types of human malignancies [84,85,86,87]. Moreover, the great majority of examined tumor tissues had reduced levels of 5hmC when compared with matched non-malignant tissues [84,85]. It has been demonstrated that decreased 5hmC is a distinctive epigenetic event that correlates with neoplastic progression in melanoma [86]. The same was true in human hepatocellular carcinoma, where the level correlated with tumor stage. Other malignancies where 5hmC and aberrant expression or mutation of TETs are linked with the disease pathogenesis are hematologic malignancies (reviewed in [88]). It is unclear how or why 5hmC is decreased in cancerous tissues. It is possible that decreased TET activity/expression is responsible [88].

Some experimental works demonstrated that 5hmC could also be deaminated by AID to yield 5hmU, which in turn may be removed by TDG or single-strand selective monofunctional uracil DNA glycosylase 1 (SMUG1) [89,90].

## 11. Pattern of DNA Epigenetic Modifications in Myeloid Malignances and MM

DNA methylation is an important regulator of hematopoiesis, and several studies suggest that disturbed DNA methylation contributes to immune disorders and hematologic malignancies (reviewed in [91,92]). *TET2* is among the most commonly mutated genes in adult myeloid malignancies, occurring in ~25% and ~50% of cases of myelodysplastic syndromes (MDS) and chronic myelomonocytic leukemia (CMML), respectively [93,94,95]. Recently, it was found that *TET2* is also mutated in MM [96]. However, whereas one third of *TET2* knockout mice developed lethal myeloid malignancies within their first year of life [97], a causative relationship between TET2 deficiency and human malignancy is less clear (reviewed in [98]). Nevertheless, the content of the epigenetic modifications mediated by TET2 (and TET1 and TET3) can be used as an indirect measure of TET dioxygenase activity. Quantification of TET-meditated DNA modifications may elucidate the consequences of TET deficiency due to *TET* mutations. The functional consequences of presumed aberrant epigenetic changes can be disease initiation and progression, the latter via interactions with other somatic mutations including those with a direct epigenetic impact or others that may have indirect effects. Recently published work has aimed to determine if there is a direct link between various mutations linked to TET activity and 5hmC, 5fC, 5caC, and 5hmU content with the goal to determine if such modifications can serve as predictive biomarkers of myeloid neoplastic disease process [99].

Accurate measurements of 5fC, 5caC, and 5hmU are challenging. The reasons for this include not only very low levels of these modified bases in mammalian genomes, but also levels of 5hmC 3–4 orders of magnitude higher, which impairs the detection and quantitation of less common metabolites. We recently developed a rapid, highly sensitive, and specific isotope-dilution automated two-dimensional ultra-performance liquid chromatography tandem mass spectrometry (2D-UPLC-MS/MS) method that is specifically tailored for simultaneous analysis of global levels of 5mC, 5hmC, 5fC, 5caC, and 5hmU (together with uracil and 8-oxodG) [100].

Using this methodology, we found that the best predictor/marker of *TET2* mutations in patients with myeloid malignances (MDS) was 5fC [99]. It is established that TETs preferred substrate is 5mC over 5hmC [101] and thus that TET predominantly forms 5hmC. However, we found that *TET2* mutations were associated with decreased 5fC and 5caC but had only a moderate influence on 5hmC [99]. By using an in vitro model, Kohli’s laboratory [102] showed that mutations in the TET2 active site scaffold (T1372-Y1902) eliminated 5fC and 5caC formation with no or only moderate influence on 5hmC formation. Our results obtained in human marrow samples add to that finding and demonstrate that several mutations located along DSBH and Cys-rich domains, which form compact catalytic domains, have a similar effect on TET2 activity. In this work, all frameshift and nonsense *TET2* mutations, likely resulting in a truncated protein, yielded similar epigenetic profiles. These results suggest that *TET2* mutations alter protein structure and function in ways that weaken its activity specifically with respect to higher order oxidation products. This work is the first in vivo evidence for a direct link between endogenously generated *TET2* mutations and the enzyme activity reflected in its product levels [99].

5fC is enriched at active enhancers involved in tissue development/differentiation [103]. This suggests that reduced levels of 5fC may be a characteristic feature of largely undifferentiated, malignant cells with *TET2* mutations. More recently, 5fC was suggested to be a key epigenetic mark controlling gene expression [104]. In sum, while previous studies of *TET* mutations focused on the 5hmC content as a first product of the dioxygenase reaction, emerging evidence suggests that levels of 5fC and 5hmU are also altered and may have pathogenetic significance. There is little consensus concerning links between TET mutations and clinical outcome. Therefore, the measurement of these alternately modified bases could potentially be helpful to improve accurate identification of the disease mechanisms and may prove valuable as prognostic and diagnostic tools in myeloid malignancies. To underline a meaning of the above described results in the context of future work, it should be mentioned that there is a relationship between MDS and MM evolution; it was found that MM patients had an 11-fold increased risk of myelodysplastic syndromes (MDS) and MGUS patients had an 8-fold increased risk of MDS [105]. A recent study employed MS quantification of global 5mC and 5hmC and also mapped 5hmC genome wide in plasma cells from 40 MM patients [106]. Here, global 5hmC was significantly decreased in myeloma compared to normal plasma cells. However, 5hmC strongly persisted on oncogenic genes such as *CCND1*, *CCND2,* and *MMSET,* as well as at enhancers of *CCND2* and *MYC*. The potential value of 5hmC as a prognostic marker was underscored by a recent study of circulating cell-free DNA from 184 newly diagnosed MM patients, in which 5hmC was mapped across an eight-gene panel and correlated with overall survival in the MM patients [107].

## 12. Conclusions

We propose that genomic uracil and aberrant profiles of demethylation intermediates might be driver events in the development of certain hematologic malignances. Intriguingly, genomic uracil and endogenously generated epigenetic DNA modifications seem to play important cellular regulatory roles (for a review see [1]); for example, the immune system of higher organisms depends upon the generation of uracil for *Ig* gene diversification. Oxidatively generated derivatives of 5mC play an important role in the active process of DNA demethylation and may have specific regulatory functions. Our review demonstrates that for DNA the terms “damage” and “modification” have different implications for the cell, but what dictates whether the presence of an altered nucleobase is defined as damage, a lesion that may lead to malignant transformation, or a modification? Genomic uracil from misincorporated dUMP is not even a modified nucleobase, unlike deaminated C. As it is a normal intermediate in dTTP biosynthesis, it is present in significant amounts in cells and is probably incorporated in substantial amounts but rapidly removed, as outlined. It cannot be excluded that such incorporation has a physiological function yet to be understood.

It is also puzzling why a potentially mutagenic DNA damage may lead to malignancy in some tissues but not in others. What processes determine the outcome? Perhaps the epigenetic DNA nucleobase modifications, e.g., 5hmC and the higher order products of TETs as well as genomic uracil are more important than previously thought since they display considerable inter-tissue differences. Moreover, transcription factors or histone modifiers that sense these modified nucleobases likely vary among tissues and developmental stages and contribute to dictate outcome. Most likely, it is not only simply the level of damage, but also its location and context that is the key factor, as discussed in some detail in a previous review [1]. The prevalence of MGUS and MM increases sharply with age and alterations of epigenome are hallmark of aging process [108]. Moreover, TET mutations were detected in the blood of elderly individuals without overt hematologic malignances [109].

The differentiation of hematopoietic stem cells toward plasmocytes (PCs) is tightly regulated by epigenetic mechanisms, and flaws in this epigenetic control can result in various B cell related disorders including MM (reviewed in [110]). There are different levels of differentiation of tumor and MM PC populations including both cells with B and PC phenotype, which in turn suggests that MM may originate either from B cells which do not complete the differentiation program or from PC that partially dedifferentiate [111]. Recently, it was found that TETs proteins have a critical role in B-cell differentiation [112]. Moreover, it was shown that TETs regulate CSR (possibly also SHM) via increasing expression of AID mRNA and protein during B cell activation [113]. Thus, an aberrant methylation/demethylation process and abnormal AID expression resulting in an increased level of genomic uracil may have profound effects on MM development.

## Figures and Tables

**Table 1:** Mammalian uracil-DNA glycosylase.

Enzyme	Subcellular Localization	Substrates and (Minor Substrates)	Mouse Knockout	Human Disease
UNG *	Nuclei and mitochondria	U, 5-FU in ss and dsDNA, U:A and U:G context (alloxan, 5-hydroxyuracil, isodialuric acid)	Partial defect in CSR, skewed SHM, B-cell lymphomas	Complete defect in CSR, HIGM syndrome, infections, lymphoid hyperplasia
SMUG1	Nuclei	5-hmU, U:G > U:A > ssU, 5-FU, εC in ss and dsDNA	Viable and fertile. SMUG1/UNG/MSH triple k.o. reduced longevity	Unknown
TDG	Nuclei	U:G > T:G, (5-hmU in dsDNA, 5-FU)	Embryonic lethal, epigenetic role in development	Unknown
MBD4	Nuclei	U:G and T:G, 5-hmU in CpG context (εC, 5-FU in dsDNA)	Viable and fertile, C to T transitions, intestinal neoplasia	Mutated in carcinomas with microsatellite instability

* The mammalian *UNG* gene encodes mRNAs for mitochondrial UNG1 and nuclear UNG2 that have identical catalytic domains but different N-terminal extensions for cellular targeting. Alternative processing of UNG1 results in the UNG1 variant that also enters nuclei and appears to support CSR. Although not specifically tested, the UNG1 variant likely has the same substrate spectrum as UNG1 and UNG2. See also references in the main text [7,8].
